# Role of clipping in aneurysmal subarachnoid hemorrhage: a post hoc analysis of the Earlydrain trial

**DOI:** 10.1007/s10143-024-03057-w

**Published:** 2024-10-26

**Authors:** Robert Mertens, Stefan Wolf, Lars Wessels, Nils Hecht, Jens Gempt, Bernhard Meyer, Florian Ringel, Veit Rohde, Peter Vajkoczy, for the Earlydrain Study Group

**Affiliations:** 1 Department of Neurosurgery, Charité – Universitätsmedizin Berlin, corporate member of Freie Universität Berlin, Humboldt-Universität zu Berlin, and Berlin Institute of Health, Berlin, Germany; 2 Center for Stroke Research Berlin, Charité – Universitätsmedizin Berlin, corporate member of Freie Universität Berlin, Humboldt-Universität zu Berlin, and Berlin Institute of Health, Berlin, Germany; 3 Berlin Institute of Health, BIH Academy, Junior Clinician Scientist Program, Charité – Universitätsmedizin Berlin, Berlin, Germany; 4 Department of Neurosurgery, University Medical Center Hamburg-Eppendorf, Hamburg, Germany; 5 Department of Neurosurgery, Klinikum rechts der Isar, Technical University Munich, Munich, Germany; 6 Department of Neurosurgery, University Medical Center Mainz, Mainz, Germany; 7 Department of Neurosurgery, University Medical Center Göttingen, Göttingen, Germany

**Keywords:** Subarachnoid hemorrhage, Cerebral aneurysm, Clipping, Coiling, Earlydrain

## Abstract

The choice between clipping and coiling of ruptured cerebral aneurysms in subarachnoid hemorrhage (SAH) remains controversial. The recently published Earlydrain trial provides the opportunity to analyze the latest clip-to-coil ratio in German-speaking countries and to evaluate vasospasm incidence and explorative outcome measures in both treatment modalities. We performed a post hoc analysis of the Earlydrain trial, a multicenter randomized controlled trial investigating the use of an additional lumbar drain in aneurysmal SAH. The decision whether to clip or to coil the ruptured aneurysm was left to the discretion of the participating centers, providing a real-world insight into current aneurysm treatment strategies. Earlydrain was performed in 19 centers in Germany, Switzerland, and Canada, recruiting 287 patients with aneurysmal SAH of all severity grades. Of these, 140 patients (49%) received clipping and 147 patients (51%) coiling. Age and clinical severity based on Hunt-Hess/WFNS grades and radiological criteria were similar. Clipping was more frequently used for anterior circulation aneurysms (55%), whereas posterior circulation aneurysms were mostly coiled (86%, p < 0.001). In high-volume recruiting centers, 56% of patients were treated with clipping, compared to 38% in other centers. A per-year analysis showed a stable and balanced clipping/coiling ratio over time. Regarding vasospasm, 60% of clipped versus 43% of coiled patients showed elevated transcranial Doppler criteria (p = 0.007), reflected in angiographic vasospasm rates (51% vs. 38%, p = 0.03). In contrast to the Earlydrain main results establishing the superiority of an additional lumbar drain, explorative outcomes after clipping and coiling measured by secondary infarctions, mortality, modified Rankin Score, Glasgow Outcome Scale Extended, or Barthel-Index showed no significant differences after discharge and at six months. In clinical practice, aneurysm clipping is still a frequently used method in aneurysmal SAH. Apart from a higher rate of vasospasm in the clipping group, an exploratory outcome analysis showed no difference between the two treatment methods. Further development of periprocedural treatment modalities for clipped ruptured aneurysms to reduce vasospasm is warranted.

## Introduction

The two predominant methods to treat cerebral aneurysms are microsurgical clipping and endovascular coiling. Whether to clip or to coil a ruptured aneurysm in subarachnoid hemorrhage (SAH) still remains a controversial debate, particularly in cases of severe SAH [1, 2]. Ruptured cerebral aneurysms should be treated as soon as possible [3–5], and the International Subarachnoid Aneurysm Trial (ISAT), published in 2002, suggested that coiling was superior to clipping in terms of independent survival for patients who were in good clinical condition and could be treated equally effectively with either clipping or coiling [6]. Further studies have shown that clipping is associated with a higher rate of vasospasm [7, 8], leading to poorer outcomes and necessitating a higher intensity of therapy [9]. All this has led to the perception that clipping will be of secondary importance in the future. While this has proven true for some centers and regions [10, 11], clipping continues to play a significant role in others [12]. The recently published Earlydrain trial, which established the superiority of an additional lumbar drain in SAH [13], offers the opportunity to analyze the latest clip-to-coil ratio in German-speaking countries. We therefore performed a post hoc analysis to provide a controlled real-world insight into current treatment strategies for ruptured cerebral aneurysms. Additionally, we aimed to evaluate the incidence of vasospasm and exploratory outcome measures in both treatment modalities.

## Methods

### Study design and oversight

This study is a post hoc analysis of the Earlydrain trial, a multicenter RCT with blinded end point evaluation performed in 19 hospitals in Germany, Switzerland, and Canada [13]. Main ethics approval was obtained from the ethics committee of the University of Erlangen, Germany, and local approval was obtained from the ethics committee at each participating center. The detailed study protocol can be found online [13]. Trial sites were centers specialized in the acute care of aneurysmal SAH, performing at least 30 aneurysm procedures per year. These centers had an interdisciplinary setup, featuring a neurovascular board with a proven track record in both state-of-the-art surgical and endovascular treatments. Patients ≥ 18 years of age with SAH on computed tomography (CT) scan and confirmation of an intracranial aneurysm by CT angiography or digital subtraction angiography (DSA) were eligible for the study and were randomized in a 1:1 ratio to receive either standard of care or additional use of a lumbar drain. Patients included in the trial were followed after randomization with documentation of baseline demographic characteristics, clinical and procedural data, descriptive and blinded radiologic imaging at discharge, and outcome after six months.

### Trial procedures and end points

Initial emergency treatment including placement of an external ventricular drain (EVD), and intubation was at the discretion of the participating centers. Aneurysm treatment had to be performed within the first 48 h after onset and aneurysms were treated with clipping or coiling, as applicable and in accordance with international guidelines and recommendations [14, 15]. The strategy of aneurysm exclusion (clipping or primary coiling) was left to the discretion of the centers and was decided by an interdisciplinary team of neurosurgeons and neuroradiologist with expertise in both surgical and endovascular treatment. The decision was based on parameters such as patient age, aneurysm size, location and configuration. Patients were included into the trial independent of this treatment decision and the decision making for aneurysm treatment was not affected by the Earlydrain study protocol. Randomization was performed after aneurysm treatment when patients returned to the intensive care unit (ICU). If patients were randomized to the lumbar drain group, a lumbar drain was placed after aneurysm treatment if a postprocedural CT scan indicated safety. A drainage rate of 5 ml per hour was started within 72 h for at least 4 days. This resulted in an equivalent of 480 ml or more of lumbar cerebrospinal fluid (CSF) drainage within the eight days to be considered as protocol-compliant treatment. Additional drainage via EVD was at the discretion of the local physicians. Primary end point of the Earlydrain Study was the rate of unfavorable neurological outcome at 6 months after SAH, measured with the modified Rankin Score (mRS) and dichotomized to either 0 to 2 (good outcome) or 3 to 6 (unfavorable outcome). Main secondary end point was the rate of secondary infarctions which were not present in the postprocedural CT scan after aneurysm treatment and were diagnosed on the final cerebral imaging (CT or magnetic resonance imaging (MRI)) prior to discharge from acute care. Further secondary end points included mortality, rate of vasospasm, requirement of a permanent ventriculo-peritoneal (VP) shunt, Barthel-Index, Glasgow Outcome Scale—Extended (GOS-E) score and mRS at discharge and at 6 months. This post hoc analysis focused on comparing patient characteristics, treatment details, and explorative outcomes after stratification for clipping versus coiling.

### Evaluation of vasospasm

Symptomatic vasospasm was monitored clinically, though detection is challenging in sedated, ventilated patients. Furthermore, vasospasm was monitored by daily transcranial Doppler (TCD; threshold: mean flow in the MCA at 50 – 60 mm depth > 120 or > 160 cm/s) and by DSA. If vasospasm was suspected or as standardized routine between day 7 to 10 after SAH onset, cerebral vascular imaging was conducted using either CT angiography, MR angiography or DSA. Angiographic vasospasm was defined as none, mild (up to 33%), moderate (34% to 66%), or severe (67% to 100%) vasoconstriction in the vasculature most affected. Treatment of confirmed vasospasm including balloon angioplasty or local application of vasodilators was not specified in the trial protocol and was at the discretion of the local investigators.

### Statistical analysis

Descriptive statistics of the patients were presented as proportions for categorical variables and means with standard deviations or medians with interquartile ranges (IQR) for continuous variables. Chi-square tests and Mann–Whitney-U tests were performed to compare parameters on nominal and ordinal scales, respectively. Explorative outcome assessment of clipping and coiling was performed with a generalized linear model. Adjustment of statistical models was performed for Hunt-Hess score > 3, age, and presence of intracerebral (ICH) and intraventricular hemorrhage (IVH), similar to the factors of outcome relevance identified in the Earlydrain main study. Odds ratios were converted to risk ratios for nuanced interpretation [16]. The significance level was set to α = 0.05. IBM SPSS version 27.0, GraphPad Prism version 10.1.1, R version 4.3.1 and RStudio version 2023.06.0 were used for statistical analysis.

## Results

### Patient characteristics

Of 287 included patients, 197 (68.6%) were female, and the median (IQR) age was 55 (48 – 63) years. A total of 140 (49%) patients were treated by clipping and 147 (51%) of patients by coiling. Table 1 demonstrates the patient characteristics stratified for clipping versus coiling. Age and clinical severity based on Hunt-Hess/WFNS grades and radiological criteria were similar. Clipping was more frequently used for aneurysms in the anterior circulation (134 of 243 patients, 55%), and the majority of posterior circulation aneurysms were coiled (38 of 44 patients, 86%, *p* < 0.001).

**Table 1:** Patient characteristics stratified for clipping vs. coiling

Characteristic	Clipping (*n* = 140)	Coiling (*n* = 147)	*p* value
**Age—years**	56 (48 to 64)	54 (47 to 63)	0.35
**Sex**			0.92
Female	97 (69.3%)	100 (68%)	
Male	43 (30.7%)	47 (32%)	
**modified Rankin Score (mRS) on admission**			0.53
0	133 (95%)	136 (92.5%)	
1	7 (5%)	11 (7.5%)	
**Hunt-Hess classification**			0.24
1	30 (21.4%)	24 (16.3%)	
2	27 (19.3%)	42 (28.6%)	
3	26 (18.6%)	33 (22.4%)	
4	23 (16.4%)	21 (14.3%)	
5	34 (24.3%)	27 (18.4%)	
**WFNS classification**			0.41
1	50 (35.7%)	45 (30.6%)	
2	19 (13.6%)	24 (16.3%)	
3	5 (3.6%)	12 (8.2%)	
4	13 (9.3%)	16 (10.9%)	
5	53 (37.9%)	50 (34%)	
**Modified Fisher classification**			0.06
1	5 (3.6%)	5 (3.4%)	
2	9 (6.4%)	3 (2%)	
3	56 (40%)	45 (30.6%)	
4	70 (50%)	94 (63.9%)	
**Intracerebral hemorrhage**	60 (42.9%)	46 (31.3%)	0.06
**Intraventricular hemorrhage**	78 (55.7%)	97 (66%)	0.1
**Aneurysm location**			** < 0.001**
ACA	15 (10.7%)	10 (6.8%)	
ACoA	37 (26.4%)	54 (36.7%)	
ICA	9 (6.4%)	15 (10.2%)	
MCA	54 (38.6%)	6 (4.1%)	
PCoA	19 (13.6%)	24 (16.3%)	
BA	1 (0.7%)	19 (12.9%)	
VA / cerebellar	5 (3.6%)	19 (12.9%)	
**Number of aneurysms**	1 (1 to 2)	1 (1 to 2)	0.38
**Size of aneurysm – mm (n = 279)**	6 (4 to 8)	6 (4 to 8)	0.39
**Aneurysm circulation**			** < 0.001**
Anterior	134 (95.7%)	109 (74.1%)	
Posterior	6 (4.3%)	38 (25.9%)	

### Treatment modalities by participating centers

**Fig. 1:**
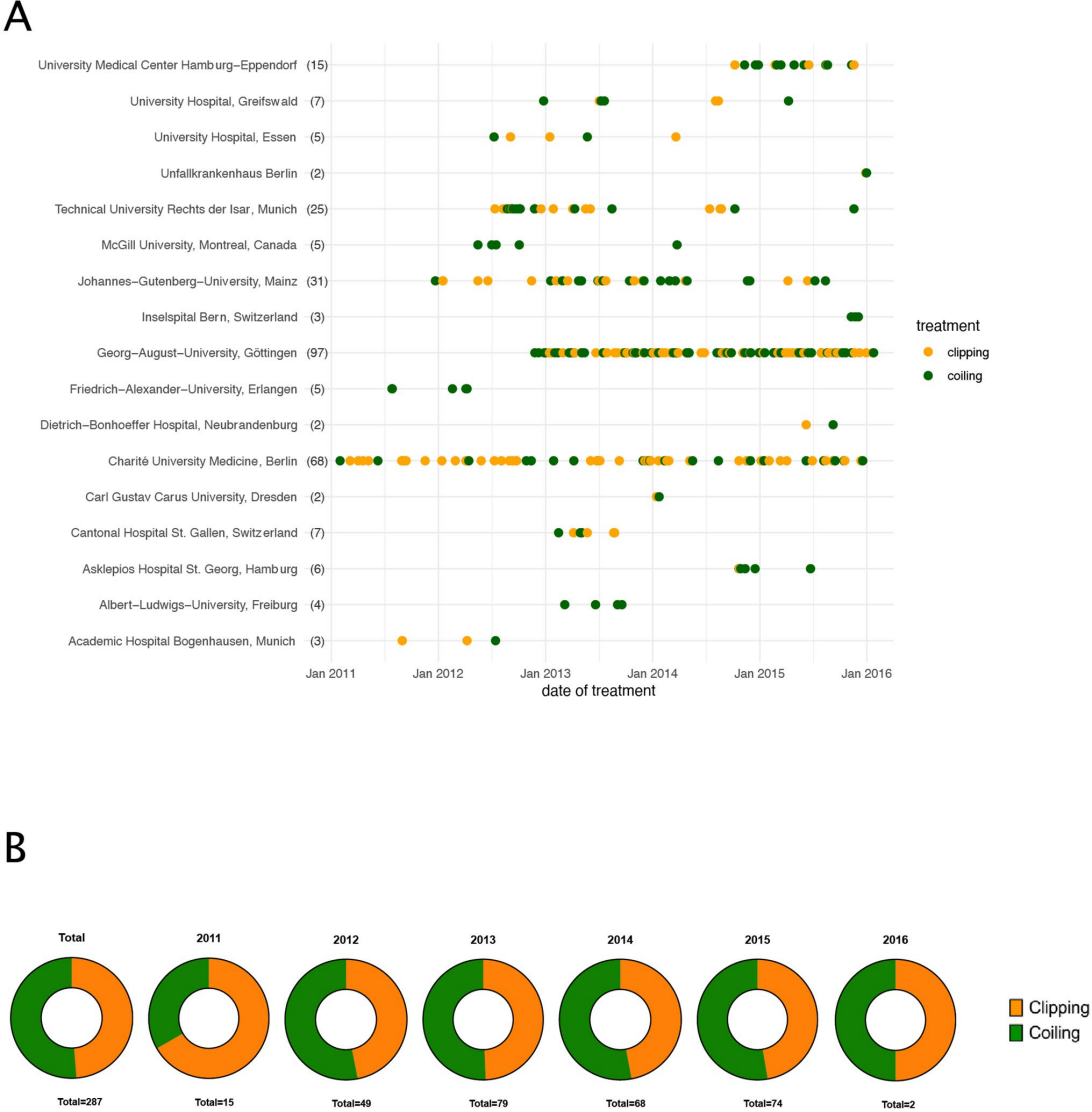
**A:** Comparison of treatment choice, stratified by clipping versus coiling, across all study centers throughout the study duration from 2011 to 2016. Each point represents a patient. **B:** Ratio of clipping versus coiling across all study centers by year

Next, we were interested whether the clip/coil relation varies among the individual centers. Figure 1A highlights the recruitment of patients, stratified by clipping versus coiling, across all participating study centers throughout the study duration. Two study centers did not recruit any patients and were therefore not listed. In high-volume recruiting centers (defined as the top 5 recruiting centers that included at least 15 patients: Göttingen, Berlin, Mainz, Munich Rechts der Isar and Hamburg Eppendorf), 53% of patients were treated with clipping, compared to 31% in all other centers. Finally, we aimed to understand whether there was a policy change in the clip versus coil decision over time (due to changes in philosophies, technical endovascular evolutions, etc.). A per-year analysis of the whole patient cohort, however, showed a stable and balanced clipping/coiling ratio over the study duration (Fig. 1B).

### Incidence of vasospasm and treatment characteristics after clipping versus coiling

The early complications after clipping and coiling of the aneurysms have already been reported elsewhere [17]. Thus, we focused on the controversy that clipping of a ruptured aneurysms might be associated with a higher incidence of vasospasm. Detailed vasospasm readouts as well as general treatment characteristics, stratified for clipping versus coiling, are listed in Table 2. One important confounder for the incidence of vasospasm could be a different CSF drainage management, leading to differences in CSF and blood washout. Importantly, the drainage amount via the lumbar drain did not differ between the clipping and coiling groups. While the rate of symptomatic vasospasm did not differ between these groups, the rate of TCD vasospasm was significantly higher in the clipping group when the threshold of mean flow in the MCA was defined > 120 cm/s (p = 0.007). If the threshold was raised to > 160 cm/s, the difference between the groups was no longer statistically significant (p = 0.06). The rates of TCD vasospasm were reflected in higher rates of angiographic vasospasm in the clipping group (51.3% vs. 38.3%, p = 0.03), primarily due to mild vasospasm (21.7% vs. 7.5%). The rates of severe angiographic vasospasm (10.4% vs 11.2%) and the use of endovascular treatment for severe vasospasm (8.6% vs 8.2%) were similar. The rate of infarctions at discharge, length of hospital stay, and discharge locations did not differ between the groups.

**Table 2:** Incidence of vasospasm and treatment characteristics stratified for clipping vs. coiling

Characteristic^1^	N (Clipping/Coiling)	Clipping (*n* = 140)	Coiling (*n* = 147)	*p* value
**Aneurysm treatment**				
Aneurysm treatment day—day after SAH	287 (140/147)	1 (0 to 1)	1 (0 to 1)	0.98
Recurrent SAH before aneurysm treatment	287 (140/147)	12 (8.6%)	5 (3.4%)	0.11
Imaging after treatment—day after treatment	287 (140/147)	1 (0 to 1)	1 (0 to 1)	0.14
**CSF drainage management**				
Start of ICU treatment—day after SAH	287 (140/147)	2 (1 to 2)	2 (1 to 2)	0.72
Patients with LD	287 (140/147)	68 (48.6%)	72 (49%)	1
Start of lumbar drainage—day after SAH	140 (68/72)	2 (1 to 2)	2 (1 to 2)	0.59
Mean daily CSF drainage via LD—ml (IQR)	140 (68/72)	106 (86 to 116)	108 (93 to 125)	0.23
CSF drainage via LD day 1–8—ml (IQR)	140 (68/72)	766 (535 to 894)	782 (558 to 920)	0.75
**Vasospasm prophylaxis**				
Vasospasm prophylaxis with nimodipine	287 (140/147)	137 (97.9%)	147 (100%)	0.23
Vasospasm prophylaxis with Mg^2+^	287 (140/147)	67 (47.9%)	89 (60.5%)	**0.04**
Vasospasm prophylaxis with statins	287 (140/147)	20 (14.3%)	26 (17.7%)	0.53
**Vasospasm assessment**				
Patients with symptomatic vasospasm	287 (140/147)	45 (32.1%)	44 (29.9%)	0.78
Patients with TCD vasospasm (> 120 cm/s)	259 (123/136)	74 (60.2%)	58 (42.6%)	**0.007**
Patients with TCD vasospasm (> 160 cm/s)	259 (123/136)	39 (31.7%)	28 (20.6%)	0.06
Day of angiographic vasospasm assessment (IQR)	222 (115/107)	8 (7 to 10)	9 (7 to 10)	0.1
Amount of angiographic vasospasm	222 (115/107)			**0.03**
No vasospasm		56 (48.7%)	66 (61.7%)	
Mild (≤ 33%)		25 (21.7%)	8 (7.5%)	
Moderate (34—66%)		22 (19.1%)	21 (19.6%)	
Severe (> 66%)		12 (10.4%)	12 (11.2%)	
Endovascular treatment for severe vasospasm	287 (140/147)	12 (8.6%)	12 (8.2%)	1
**Hospital stay**				
Days in acute hospital (IQR)	287 (140/147)	24 (19 to 31)	23 (18 to 32)	0.56
Days from SAH to last imaging before discharge (IQR)	287 (140/147)	18 (11 to 25)	18 (11 to 26)	0.91
Infarct at discharge	287 (140/147)	52 (37.1%)	46 (31.3%)	0.36
Discharge location	287 (140/147)			0.75
Home		35 (25%)	43 (29.3%)	
Rehabilitation		75 (53.6%)	78 (53.1%)	
Other hospital		11 (7.9%)	8 (5.4%)	
Died in acute hospital		19 (13.6%)	18 (12.2%)	
Interview of survivors at day 180	240 (116/124)			0.11
Patient		75 (64.7%)	95 (76.6%)	
Relative		32 (27.6%)	21 (16.9%)	
Healthcare professional		9 (7.8%)	8 (6.5%)	

^1^Data are median (IQR) or n (%)

### Explorative outcome analysis stratified for clipping versus coiling

We performed an explorative analysis of clinical outcomes stratified for clipping versus coiling (Table 3). Concerning the primary endpoint of the Earlydrain trial, the rates of mRS grades of 3 to 6 after 6 months were numerically higher in the clipping group (44.3% vs. 33.3%), but no significance was reached, neither in the uncorrected analysis (p = 0.06) nor in the severity-adjusted analysis for age, Hunt-Hess grade > 3, ICH and IVH (p = 0.13). Similar, other outcome parameters after discharge and at 6 months, such as requirement of VP-shunt, mortality, GOS-E and Barthel-Index, did not demonstrate any significant differences between the groups.

**Table 3:** Explorative clinical outcome stratified for clipping vs. coiling. Severity adjustment of statistical model was performed for Hunt-Hess grade > 3, age, and presence of intracerebral and intraventricular hemorrhage

Variable	Clipping (*n* = 140)	Coiling (*n* = 147)	Absolute risk difference clipping—coiling (95% CI)	Relative Risk for clipping (95% CI)	*p* value	Severity adjusted Relative Risk (95% CI)	*p* value
**Vasospasm**							
Patients with clinically suspected vasospasm	45/140 (32.1%)	44/147 (29.9%)	0.02 (-0.09 to 0.13)	1.07 (0.75 to 1.47)	0.69	1.11 (0.77 to 1.51)	0.56
Patients with TCD vasospasm (> 120 cm/s)	74/123 (60.2%)	58/136 (42.6%)	0.18 (0.06 to 0.3)	1.41 (1.13 to 1.67)	**0.005**	2.07 (1.39 to 2.92)	** < 0.001**
Patients with TCD vasospasm (> 160 cm/s)	39/123 (31.7%)	28/136 (20.6%)	0.11 (0.01 to 0.22)	1.54 (1.02 to 2.19)	**0.04**	1.66 (1.12 to 2.26)	**0.014**
Any angiographic vasospasm	59/115 (51.3%)	41/107 (38.3%)	0.13 (0 to 0.26)	1.34 (1.0 to 1.68)	0.05	1.34 (1.0 to 1.67)	0.05
**VP-Shunt**							
Requirement of VP-shunt during acute care	31/140 (22.1%)	37/147 (25.2%)	-0.03 (-0.13 to 0.07)	0.88 (0.56 to 1.31)	0.55	0.88 (0.56 to 1.31)	0.55
Requirement of VP-shunt at 6 months	39/140 (27.9%)	44/147 (29.9%)	-0.02 (-0.14 to 0.08)	0.93 (0.63 to 1.31)	0.7	0.84 (0.55 to 1.2)	0.37
**Mortality**							
Mortality at discharge	19/140 (13.6%)	18/147 (12.2%)	0.01 (-0.06 to 0.09)	1.11 (0.59 to 1.96)	0.74	1.01 (0.64 to 1.59)	0.78
Mortality at 6 months	22/140 (15.7%)	22/147 (15%)	0.01 (-0.08 to 0.09)	1.05 (0.6 to 1.75)	0.86	0.98 (0.59 to 1.47)	0.93
**Modified Rankin Score (mRS)**							
mRS 3–6 at discharge	97/140 (69.3%)	92/147 (62.6%)	0.07 (-0.04 to 0.18)	1.11 (0.93 to 1.26)	0.23	1.14 (0.77 to 1.55)	0.48
mRS 3–6 at 6 months	62/140 (44.3%)	49/147 (33.3%)	0.11 (0 to 0.22)	1.33 (0.99 to 1.69)	0.06	1.27 (0.93 to 1.62)	0.13
**Glasgow Outcome Scale—Extended (GOS-E)**							
GOS-E 1–4 at discharge	88/140 (62.9%)	83/147 (56.5%)	0.07 (-0.05 to 0.18)	1.11 (0.91 to 1.3)	0.27	1.09 (0.73 to 1.49)	0.66
GOS-E 1–4 at 6 months	48/140 (34.3%)	44/147 (29.9%)	0.04 (-0.06 to 0.15)	1.15 (0.81 to 1.54)	0.43	1.02 (0.67 to 1.42)	0.93
**Barthel-Index**							
Barthel-Index ≤ 80 at discharge	75/121 (62%)	69/129 (53.5%)	0.09 (-0.04 to 0.21)	1.16 (0.93 to 1.37)	0.18	1.14 (0.76 to 1.56)	0.49
Barthel-Index ≤ 80 at 6 months	31/117 (26.5%)	24/124 (19.4%)	0.07 (-0.04 to 0.18)	1.37 (0.85 to 2.06)	0.19	1.2 (0.78 to 1.65)	0.38

## Discussion

Our post hoc analysis of the Earlydrain trial highlights three main findings. First, a per-year analysis revealed a consistent and balanced clipping/coiling ratio over the study duration. Accordingly, the Earlydrain trial as one of the most recent RCTs in SAH demonstrates that aneurysm clipping is still a frequently applied method in clinical practice across German-speaking countries. Second, explorative outcome rates after clipping and coiling, other than the rate of vasospasm, showed no statistically significant differences between the groups. Third, rates of TCD and angiographic vasospasm were higher after microsurgical clipping.

While the incidence of SAH has remained stable, the volume of endovascular treatment increased significantly in ruptured and non-ruptured aneurysms in the United States of America (USA) from 1993 to 2015 according to the Nationwide Inpatient Sample (NIS) database, whereas clipping decreased for ruptured aneurysms and slightly increased for non-ruptured aneurysms [10]. Shah et al*.* reported regional differences with a higher prevalence of surgical clipping in the West of the USA compared to other regions (18.8 vs. 11.6%; p < 0.0001) from 2009 to 2018 according to the Vizient Clinical Data Base, and surgical clipping was used in 13,866 (12.7%) patients, whereas endovascular coiling was used in 11,146 (10.2%) patients of the total cohort of 109,034 patients with primary ICD diagnosis of non-traumatic SAH (including non-aneurysmal SAH) [12]. Reports from Europe are limited to a few single-center studies, which showed a rapid shift to endovascular treatment nearly a decade later than in the USA [11, 18, 19]. To determine whether progressive technical developments in the endovascular field, including an expanding variety of coils, influenced treatment choices in mainly German-speaking countries during the study duration, we performed a per-year analysis of the Earlydrain cohort which showed a stable and balanced clipping/coiling ratio. According to these findings, aneurysm clipping is still a frequently used method in aneurysmal SAH. Of note, our data only extends up to the year 2016 and this ratio may have changed given the further developments since then. Data on patients requiring stent-assisted coiling was not available in our study, as these patients were excluded per protocol due to required double-platelet anticoagulation which prohibited insertion of a lumbar drain. Nevertheless, evidence supporting the use of stent-assisted coiling in SAH is limited and higher hemorrhagic and complication rates compared to coiling alone have been reported [20, 21]. Intrasaccular devices and coated stents that do not require dual anti-platelet therapy have only been developed and broadly applied in SAH within the last 5 years and were therefore not included in the study. The influence of these newer techniques on current practice has yet to be determined.

Our explorative outcome analysis of real-world clinical practice in predominantly German-speaking countries shows no significant differences in outcomes between the clipping and the coiling groups regarding mRS, infarction at discharge, requirement of VP-shunt, mortality, GOS-E or Barthel-Index. A recent Cochrane review mainly based on the ISAT trial stated, that for patients in good clinical condition with ruptured aneurysms of either the anterior or posterior circulation, coiling is associated with a better outcome if the aneurysm is suitable for both treatment methods [2]. During the recruitment of ISAT, only 22% of SAH patients treated at the participating centers were enrolled in the study. Furthermore, patients in poor clinical condition were underrepresented in the ISAT cohort as 88% of included patients were in good clinical condition (WFNS Grade 1–2) [6]. Thus, there is limited treatment evidence from RCTs that can be applied to SAH patients with poor clinical condition [2]. Furthermore, the advantage of coiling over clipping cannot be assumed for patients under 40 years old according to a post hoc analysis of the ISAT results [22]. The treatment choice should consider various factors, such as clinical condition, location and configuration of the aneurysm, as well as the skill level of the surgeon. Particularly for younger patients and those with aneurysms in certain locations, such as the middle cerebral artery (MCA), surgical clipping may still be the favorable treatment option due to its lower risk of aneurysm recurrence, rebleeding, and the need for retreatment [23, 24]. The ISAT trial randomized ruptured intracranial aneurysms considered to be suitable for both, microsurgical and endovascular treatment [6]. In contrast, aneurysms were treated according to the discretion of the local centers in the Earlydrain trial [13], constituting a substantial difference between these studies. Of note, our post hoc analysis does not have the same evidence level as prospective RCTs comparing clipping and coiling. Nevertheless, interdisciplinary treatment decisions as applied in the Earlydrain trial, in which advantages and risks of both treatment modalities were considered equally, represent a realistic and accurate scenario of real-world clinical practice. Our resulting exploratory outcome analysis included a representative patient cohort regarding WFNS and Hunt-Hess grades without differences between the clipping and coiling group, and clinical outcomes were without statistically significant difference. A meta-analysis and systematic review of three RCTs and 37 observational studies including ISAT [6] and the Barrow Ruptured Aneurysm Trial (BRAT) [25] (which has been excluded from the Cochrane Review) reported a better life quality (mRS 0–2) as well as a lower incidence of postprocedural complications after coiling, and a lower rate of mortality, rebleeding, hydrocephalus, and a higher rate of complete aneurysm occlusion after clipping [7]. Higher re-rupture rates after coiling were also reported in another recent meta-analysis (1.5% vs. 0.5%, p = 0.002; median follow-up of 6.1 years) [26]. Yet, properly designed and conducted RCTs are necessary to determine the optimal treatment strategy for intracranial aneurysms and to settle this ongoing controversy [27].

The only significant difference between the groups was the rate of vasospasm. At total of 60.2% of clipped versus 42.6% of coiled patients showed signs of elevated TCD criteria (p = 0.007). Notably, TCD measurements come with limitations, including unreliability and variability across different examiners [28–30]. TCD is also technically easier and more reliable in the anterior compared to the posterior circulation [28, 29]. Since the coiling group had a higher proportion of patients with posterior circulation aneurysms, there is a risk of underestimating TCD vasospasm in this group, constituting a notable limitation when comparing the clipping and coiling groups. Nevertheless, this finding was reflected in the higher rates of angiographic vasospasm in the clipping group and has been confirmed in the meta-analysis conducted by Peng et al*.* which analyzed 13 publications with a total of 2857 patients regarding vasospasm. Here, postprocedural vasospasm occurred less frequently in the coiling than in the clipping group (20.5% vs. 24.8%; OR = 0.787; CI = 0.649 – 0.954; p < 0.05) [7]. Furthermore, a meta‐analysis conducted by Zhu et al*.* showed a 45% increase in the risk of vasospasm if patients were treated by clipping (RR: 1.45, 95% CI: 1.23 – 1.71, p < 0.001) [8]. Angiographic vasospasm contributes to poor outcomes [9]. Consequently, further development of periprocedural treatment modalities for clipped aneurysms in SAH to reduce vasospasm is warranted. Promising results have been reported, for example, in a randomized controlled phase IIA/B study assessing the safety and tolerability of NicaPlant®, a modified prolonged release formulation of the calcium channel blocker nicardipine. In this approach, nicardipine implants are applied locally in patients with aneurysmal SAH undergoing microsurgical clipping, potentially reducing systemic side effects and improving outcomes. The placement of nicardipine implants during clipping raised no safety concerns [31], and a recently published RCT confirmed that this approach can safely and effectively prevent vasospasm after aneurysmal SAH [32]. Given these promising results, a phase III clinical trial to investigate the impact on clinical outcomes may now be justified.

### Limitations

The Earlydrain trial has not been designed for a direct comparison between clipping and coiling, and the statistical analyses should therefore be treated with caution. As a consequence, the explorative outcome analysis may lack sufficient statistical power and does not claim to answer the question whether aneurysms should be clipped or coiled. Yet our data shows that a careful interdisciplinary treatment decision can produce a balanced clinical outcome. Another limitation is the lack of long-term outcome data exceeding 6 months, which could offer a more thorough comprehension of the therapeutic impact over an extended duration. Nevertheless, the Earlydrain study is one of the most recently published multicenter RCT in SAH and the data provide a representative real-world scenario of clinical practice regarding clipping versus coiling in SAH.

## Conclusion

This post hoc analysis of the Earlydrain trial highlights that clipping of ruptured aneurysms remains a frequently applied method in the treatment of aneurysmal subarachnoid hemorrhage. The increased occurrence of vasospasms following microsurgical clipping necessitates the development of peri-clipping techniques to reduce vasospasm. In contrast to the Earlydrain main results establishing the superiority of using an additional lumbar drain after aneurysmal subarachnoid hemorrhage, explorative outcome rates after clipping and coiling measured by secondary infarctions, mortality and the modified Rankin score at six months were without statistically significant difference.

## Data Availability

The Earlydrain data supporting the conclusions presented herein is available at the Mendeley repository. (https://data.mendeley.com/datasets/c4jc7k5ptx/1).

## Abbreviations

BRAT: Barrow Ruptured Aneurysm Trial
CSF: Cerebrospinal fluid
CT: Computed tomography
DCI: Delayed cerebral ischemia
DSA: Digital subtraction angiography
EVD: External ventricular drain
GOS-E: Glasgow outcome scale extended
ICH: Intracerebral hemorrhage
ICU: Intensive care unit
IQR: Interquartile range
ISAT: International subarachnoid aneurysm trial
IVH: Intraventricular hemorrhage
MCA: Middle cerebral artery
MRI: Magnetic resonance imaging
mRS: Modified Rankin Score
NIS: Nationwide Inpatient Sample
RCT: Randomized controlled trial
SAH: Subarachnoid hemorrhage
TCD: Transcranial Doppler
USA: United States of America
VP: Ventriculo-peritoneal
WFNS: World Federation of Neurosurgical Societies

## References

[1] Ikawa F et al (Apr.2020) In-hospital mortality and poor outcome after surgical clipping and endovascular coiling for aneurysmal subarachnoid hemorrhage using nationwide databases: a systematic review and meta-analysis. Neurosurg Rev 43(2):655–667. 10.1007/s10143-019-01096-2 30941595 10.1007/s10143-019-01096-2

[2] Lindgren A et al (2018) Endovascular coiling versus neurosurgical clipping for people with aneurysmal subarachnoid haemorrhage. Cochrane Database Syst Rev 8(8):CD003085. 10.1002/14651858.CD003085.pub3 30110521 10.1002/14651858.CD003085.pub3 PMC6513627

[3] Neifert SN et al (Jun.2021) Aneurysmal Subarachnoid Hemorrhage: the Last Decade. Transl Stroke Res 12(3):428–446. 10.1007/s12975-020-00867-0 33078345 10.1007/s12975-020-00867-0

[4] Hostettler IC et al (2023) Duration between aneurysm rupture and treatment and its association with outcome in aneurysmal subarachnoid haemorrhage. Sci Rep 13(1):1527. 10.1038/s41598-022-27177-9 36707604 10.1038/s41598-022-27177-9 PMC9883503

[5] van Lieshout JH et al (Sep.2022) Development and Internal Validation of the ARISE Prediction Models for Rebleeding After Aneurysmal Subarachnoid Hemorrhage. Neurosurgery 91(3):450–458. 10.1227/neu.0000000000002045 35881023 10.1227/neu.0000000000002045

[6] Molyneux A et al (Oct.2002) International Subarachnoid Aneurysm Trial (ISAT) of neurosurgical clipping versus endovascular coiling in 2143 patients with ruptured intracranial aneurysms: a randomised trial. Lancet 360(9342):1267–1274. 10.1016/s0140-6736(02)11314-6 12414200 10.1016/s0140-6736(02)11314-6

[7] Peng C, Diao YH, Cai SF, Yang XY (2022) Endovascular coiling versus microsurgical clipping for ruptured intracranial aneurysms: a meta-analysis and systematic review. Chin Neurosurg J 8(1):17. 10.1186/s41016-022-00283-3 35879784 10.1186/s41016-022-00283-3 PMC9310462

[8] Zhu W, Ling X, Petersen JD, Liu J, Xiao A, Huang J (Apr.2022) Clipping versus coiling for aneurysmal subarachnoid hemorrhage: a systematic review and meta-analysis of prospective studies. Neurosurg Rev 45(2):1291–1302. 10.1007/s10143-021-01704-0 34870768 10.1007/s10143-021-01704-0 PMC8976818

[9] Weir B (Oct.2020) Vasospasm: does it cause infarction and poor outcome? J Neurosurg 134(3):1006–1011. 10.3171/2020.7.JNS202551 33126209 10.3171/2020.7.JNS202551

[10] Golnari P et al (Feb.2020) Volumes, outcomes, and complications after surgical versus endovascular treatment of aneurysms in the United States (1993–2015): continued evolution versus steady-state after more than 2 decades of practice. J Neurosurg 134(3):848–861. 10.3171/2019.12.JNS192755 32032946 10.3171/2019.12.JNS192755

[11] Calvanese F et al (2024) Changes in treatment of intracranial aneurysms during the last decade in a large European neurovascular center. Acta Neurochir 166(1):173. 10.1007/s00701-024-06064-4 38594469 10.1007/s00701-024-06064-4 PMC11004042

[12] Shah VA et al (2022) Regional Variability in the Care and Outcomes of Subarachnoid Hemorrhage Patients in the United States. Front Neurol 13:908609. 10.3389/fneur.2022.908609 35785364 10.3389/fneur.2022.908609 PMC9243235

[13] Wolf S et al (2023) Effectiveness of Lumbar Cerebrospinal Fluid Drain Among Patients With Aneurysmal Subarachnoid Hemorrhage: A Randomized Clinical Trial. JAMA Neurol 80(8):833–842. 10.1001/jamaneurol.2023.1792 37330974 10.1001/jamaneurol.2023.1792 PMC10277935

[14] Diringer MN et al (Sep.2011) Critical care management of patients following aneurysmal subarachnoid hemorrhage: recommendations from the Neurocritical Care Society’s Multidisciplinary Consensus Conference. Neurocrit Care 15(2):211–240. 10.1007/s12028-011-9605-9 21773873 10.1007/s12028-011-9605-9

[15] Connolly ESJ et al (Jun.2012) Guidelines for the management of aneurysmal subarachnoid hemorrhage: a guideline for healthcare professionals from the American Heart Association/american Stroke Association. Stroke 43(6):1711–1737. 10.1161/STR.0b013e3182587839 22556195 10.1161/STR.0b013e3182587839

[16] Cummings P (May2009) The relative merits of risk ratios and odds ratios. Arch Pediatr Adolesc Med 163(5):438–445. 10.1001/archpediatrics.2009.31 19414690 10.1001/archpediatrics.2009.31

[17] Früh A, Wolf S, Wasilewski D, Vajkoczy P, Truckenmueller P (Apr.2024) Early Complications and Outcome After Treatment of Ruptured Aneurysms in Patients with Subarachnoid Hemorrhage-A Post Hoc Analysis of the EARLYDRAIN Trial. World Neurosurg 184:e720–e730. 10.1016/j.wneu.2024.02.018 38340802 10.1016/j.wneu.2024.02.018

[18] Korja M, Lehto H, Juvela S, Kaprio J (Sep.2016) Incidence of subarachnoid hemorrhage is decreasing together with decreasing smoking rates. Neurology 87(11):1118–1123. 10.1212/WNL.0000000000003091 27521438 10.1212/WNL.0000000000003091 PMC5027805

[19] Salaud C, Hamel O, Riem T, Desal H, Buffenoir K (Feb.2016) Management of aneurysmal subarachnoid haemorrhage with intracerebral hematoma: Is there an indication for coiling first? Study of 44 cases. Interv Neuroradiol 22(1):5–11. 10.1177/1591019915617320 26634802 10.1177/1591019915617320 PMC4757382

[20] Bsat S et al (Oct.2020) Safety of stent-assisted coiling for the treatment of wide-necked ruptured aneurysm: A systematic literature review and meta-analysis of prevalence. Interv Neuroradiol 26(5):547–556. 10.1177/1591019920945059 32741229 10.1177/1591019920945059 PMC7645187

[21] Nabizadeh F, Valizadeh P, Balabandian M (Jan.2024) Stent-assistant versus non-stent-assistant coiling for ruptured and unruptured intracranial aneurysms: A meta-analysis and systematic review. World Neurosurg X 21:100243. 10.1016/j.wnsx.2023.100243 38221954 10.1016/j.wnsx.2023.100243 PMC10787302

[22] Mitchell P, Kerr R, Mendelow AD, Molyneux A (Mar.2008) Could late rebleeding overturn the superiority of cranial aneurysm coil embolization over clip ligation seen in the International Subarachnoid Aneurysm Trial? J Neurosurg 108(3):437–442. 10.3171/JNS/2008/108/3/0437 18312088 10.3171/JNS/2008/108/3/0437

[23] Hulsbergen AFC et al (Dec.2019) Long-Term Durability of Open Surgical versus Endovascular Repair of Intracranial Aneurysms: A Systematic Review and Meta-Analysis. World Neurosurg 132:e820–e833. 10.1016/j.wneu.2019.08.002 31419590 10.1016/j.wneu.2019.08.002

[24] Molyneux AJ, Birks J, Clarke A, Sneade M, Kerr RSC (Feb.2015) The durability of endovascular coiling versus neurosurgical clipping of ruptured cerebral aneurysms: 18 year follow-up of the UK cohort of the International Subarachnoid Aneurysm Trial (ISAT). Lancet 385(9969):691–697. 10.1016/S0140-6736(14)60975-2 25465111 10.1016/S0140-6736(14)60975-2 PMC4356153

[25] Spetzler RF et al (Sep.2015) The Barrow Ruptured Aneurysm Trial: 6-year results. J Neurosurg 123(3):609–617. 10.3171/2014.9.JNS141749 26115467 10.3171/2014.9.JNS141749

[26] Wach J, Vychopen M, Güresir A, Guranda A, Nestler U, Güresir E (2024) A Long-Term Comparative Analysis of Endovascular Coiling and Clipping for Ruptured Cerebral Aneurysms: An Individual Patient-Level Meta-Analysis Assessing Rerupture Rates. J Clin Med 13(6). 10.3390/jcm13061778 10.3390/jcm13061778 PMC10970744 38541999

[27] Darsaut TE, Kotowski M, Raymond J (2012) How to choose clipping versus coiling in treating intracranial aneurysms. Neurochirurgie 58(2–3):61–75. 10.1016/j.neuchi.2012.02.023 22483173 10.1016/j.neuchi.2012.02.023

[28] Wabl R (2024) On Using the Wrong Tool: Transcranial Doppler to Screen for Large Vessel Vasospasm After Aneurysmal Subarachnoid Hemorrhage. Crit Care Med. 10.1097/CCM.0000000000006262 10.1097/CCM.0000000000006262 38488421

[29] Mastantuono J-M, Combescure C, Elia N, Tramèr MR, Lysakowski C (Oct.2018) Transcranial Doppler in the Diagnosis of Cerebral Vasospasm: An Updated Meta-Analysis. Crit Care Med 46(10):1665–1672. 10.1097/CCM.0000000000003297 30080684 10.1097/CCM.0000000000003297

[30] Hollingworth M, Jamjoom AAB, Bulters D, Patel HC (2019) How is vasospasm screening using transcranial Doppler associated with delayed cerebral ischemia and outcomes in aneurysmal subarachnoid hemorrhage? Acta Neurochir (Wien) 161(2):385–392. 10.1007/s00701-018-3765-8 30637487 10.1007/s00701-018-3765-8

[31] Kerschbaumer J et al (2023) A randomized, single ascending dose safety, tolerability and pharmacokinetics study of NicaPlant® in aneurysmal subarachnoid hemorrhage patients undergoing clipping. Brain Spine 3:102673. 10.1016/j.bas.2023.102673 38021019 10.1016/j.bas.2023.102673 PMC10668089

[32] Wessels L et al (2024) Localized Nicardipine Release Implants for Prevention of Vasospasm After Aneurysmal Subarachnoid Hemorrhage: A Randomized Clinical Trial. JAMA Neurol. 10.1001/jamaneurol.2024.2564 10.1001/jamaneurol.2024.2564 PMC11334004 39158893

